# Assessment of a Digital Health Platform Using Web Analytics and User Experience Measurements: Quantitative Study Based on RE-AIM

**DOI:** 10.2196/64903

**Published:** 2026-07-02

**Authors:** Lior Weinreich, Louisa-Marie von Kontz, Björn Witzel, Olga Hermansson, Hanna Laura Hampe, Susanne Volkmer, Gerd Schulte-Körne, Kristina Moll

**Affiliations:** 1 Department of Child and Adolescent Psychiatry, Psychosomatics and Psychotherapy Faculty of Medicine Ludwig Maximilian University of Munich University Hospital Munich Germany; 2 Department of Teaching and Educational Technology University of Zurich Zurich Switzerland

**Keywords:** data science, digital health, learning disorders, Reach, Effectiveness, Adoption, Implementation, and Maintenance, RE-AIM, usability testing

## Abstract

**Background:**

In recent years, the field of digital health has grown exponentially, leading to notable benefits, such as easier access to health-related information, but also to content saturation and misinformation. Thus, it is crucial to identify digital health tools that provide meaningful value and assess them in real-world settings.

**Objective:**

This preregistered study aims to quantitatively assess the Lernstörungen Online-Diagnostik und Intervention (LONDI) platform, a German platform designed for different user groups supporting children with learning disorders. This assessment focused on user groups of mental health professionals (ie, learning therapists and school psychologists) and was grounded in 4 of the 5 reach, effectiveness, adoption, implementation, and maintenance (RE-AIM) framework dimensions: reach, adoption, implementation, and maintenance.

**Methods:**

Data were collected over a 10-month period between May 1, 2024, and March 1, 2025. The reach dimension was measured via a pop-up questionnaire (n=1324) collecting demographic and professional experience data. The adoption dimension was measured via a second pop-up questionnaire (n=160) measuring user experience (UX) and reuse intention for the platform’s help system. The implementation dimension was measured via web analytics (N=37,133) measuring reading time for pages intended for mental health professionals. Moreover, this dimension was also assessed by comparing chatbot engagement rates with industry benchmarks, in the absence of established benchmarks for digital health chatbots. The maintenance dimension was measured via web analytics as well, comparing the use in the previous (n=20,496) and current platform versions (N=37,133) in terms of number and location of users, time spent on the platform, number of actions per visit, and used devices and software.

**Results:**

A total of 21.90% (291/1324) and 10.64% (141/1324) of the users who filled out the first pop-up questionnaire stated that they were learning therapists or school psychologists, respectively, exceeding their percentage in the German population (<0.01%). The second pop-up questionnaire revealed an overall mean UX score of 1.54 (SD 1.14), surpassing the benchmark average, and UX ratings predicted intention to reuse. Time spent on the pages intended for mental health professionals was below the time needed to read them. The 0.18% rate of chatbot engagement was very low compared with industry benchmarks of 35% to 40%. Use changed in the 2 time periods compared, and most strikingly, there was an 81.2% (n=16,637) increase in the number of users.

**Conclusions:**

The study provides evidence of the LONDI platform’s positive contribution to public health in terms of the reach, adoption, and maintenance dimensions of the RE-AIM framework. Further research and endeavors are needed to better understand and improve the platform’s contribution in terms of the implementation dimension.

## Introduction

In recent years, the constant growth in digitalization has led to an exponential growth in digital health [1]. Digital health is a term encompassing various digital technologies aiming to improve health-related knowledge and practices [2]. One report estimated that in 2024, the number of digital health apps reached 337,000 [3]. Other reports estimated that in 2024, investments in American companies offering digital health tools exceeded $10.1 billion [4,5]. In particular, investments in artificial intelligence (AI)–operated digital health tools have been on the rise [4]. Specifically, AI-operated chatbots have become increasingly popular, as they are often cost-effective and provide users with 24/7 support, potentially replacing the long waiting times required for human responses [6,7]. The expansion of digital health has led to several notable benefits, such as easier access to health-related information, automation of time-consuming processes, facilitation of diagnostic and intervention decisions, and reaching reluctant populations [1,8,9]. For example, it has been found that while adolescents are often reluctant to seek traditional mental health services, they are interested in using anonymous online mental health resources [9,10]. Overall, digital health has the potential to contribute to a healthier society [1].

Misinformation and misconceptions are common in many health-related fields. For example, although research has shown that learning disorders (LDs) are caused by a complex interplay between brain development, genetics, and environmental factors [11], myths about their origin are still commonly believed (eg, LDs are caused by laziness [12]). Other myths pertaining to LDs are that people with LDs are less intelligent, children with LDs outgrow them in adulthood, and that LDs impact everyone in the same way [12]. Although the rise in digitalization positively contributes to children and adolescents’ health-related knowledge, it also leads to content saturation, making it harder to distinguish between trustworthy and inaccurate information [13]. Moreover, adults as well as adolescents often turn to social media platforms such as YouTube and Facebook for health-related information, where they are often misinformed [13,14]. To reduce misinformation and misconceptions, health professionals and academic institutions are encouraged to increase their online presence [14]. Furthermore, it is imperative to identify digital health tools that provide actual positive value by rigorously assessing them [15].

One notable assessment framework for public health outcomes is the reach, effectiveness, adoption, implementation, and maintenance (RE-AIM) framework [16]. For over 2 decades, numerous studies have used either all or some of the RE-AIM dimensions to evaluate health promotion interventions [17-19]. Specifically, the reach dimension refers to the proportion of reached users from a targeted population, effectiveness refers to an intervention’s success rate, adoption refers to the proportion of users from a targeted population planning to adopt an intervention, implementation refers to the manner in which an intervention is implemented in real-world settings, and maintenance refers to long-term sustainability [16]. Although these dimensions were initially designed to assess health promotion interventions, they have been used in recent years in other health-related contexts [20]. For example, Fuller et al [21] used RE-AIM to evaluate a digital health tool to facilitate hospital discharge preparation.

In the context of digital health, notable means of assessment are web analytics and user experience (UX) measurements. In terms of web analytics, one example is the GDPR (General Data Protection Regulation) compliant software Matomo [22]. Matomo can be used to track a plethora of anonymous user data, such as the time users spend on different pages, as well as the device and browser they are using [23-25]. This makes it possible to estimate if users spend enough time on a page to read its content, based on words per minute (WPM) calculations [26,27]. Moreover, web analytics can be used to track human-machine engagement rates. These rates can then be used to assess the quality of UX [28]. Nevertheless, while it is possible to gain valuable insights using web analytics, these insights are limited. Specifically, it is not possible to infer users’ understanding, intentions, or attitudes. Therefore, web analytics can be complemented by self-reported measures (eg, questionnaires evaluating chatbot-related attitudes [29,30]). Similarly, in terms of UX measurements, these include tracking user messages [31], as well as self-reported measures [32-34]. This makes it possible to evaluate different UX facets such as usability (ie, ease of use), perceived value, credibility, and satisfaction [35]. Taken together, a multimodal data collection process combining web analytics and self-reported measures can lead to detailed insights into evaluations of digital health platforms [36].

Lernstörungen Online-Diagnostik und Intervention (LONDI) is a German digital health platform that provides evidence-based information on LDs, as well as an algorithm-based help system for professionals to select suitable diagnostic tools and intervention programs [37]. The platform was designed for the specific needs of different user groups supporting children with LDs, namely parents, teachers, school psychologists, learning therapists, and social workers. The content featured on the platform is based on scientific findings and was created by researchers from 2 German academic institutes (the Ludwig Maximilian University Hospital and the Leibniz Institute for Research and Information in Education). The informational part of the platform is different for each user group, to accommodate the specific needs identified by the researchers after consulting with different user group representatives (eg, regulatory information that is only relevant for social workers appears on the information page designated to them). The help system part of the platform was designed for 2 of the user groups: school psychologists and learning therapists. These mental health professionals can use the help system to get diagnostic and intervention recommendations according to each child’s individual learning profile. Thus, the overall purpose of the LONDI platform is to alleviate the hardship experienced by children with LDs by providing information and resources to the relevant user groups supporting them.

The work on LONDI began in 2017, with the plan to conduct 2 evaluation phases, identify key issues after each phase, and revise the platform accordingly. The first phase assessed the initial version of LONDI, which was launched in 2022. This phase included 2 preregistered studies. The first was a quantitative study evaluating anonymous online use in real-world settings [23]. The second was a mixed methods study evaluating input from parents of children with LDs and learning therapists using LONDI in guided sessions [38]. The main issue identified in the first study was that with time, more users were accessing LONDI via smartphone devices, whereas in the first months after its launch, most users were accessing LONDI via desktop devices. This was problematic since at that time, LONDI was not optimized for smartphone use. The main issues identified in the second study were that many users disliked the platform’s “text-heavy” content and that the help system was too complicated. Therefore, the newest revised platform version was launched in 2024, after the following changes were made: LONDI was optimized for smartphone use, the platform was redesigned to feature more infographics (ie, graphic visual representations of information), the help system’s design was simplified, and instructional tutorials were added. Furthermore, in alignment with the increase in the integration of chatbots (ie, AI-based conversational proxies) in digital health platforms [6], a simple chatbot was added. As noted by Abd-Alrazaq et al [39], evaluating chatbot performance can be a useful outcome metric to assess mental health interventions. Additionally, further resources were devoted to increasing the platform’s social media presence (ie, by regularly posting on the LONDI Instagram and Facebook accounts).

The current preregistered study is part of the second LONDI evaluation phase. Its goal was to quantitatively evaluate the anonymous online use of the revised LONDI version (Table S1 in Multimedia Appendix 1 provides the specific elements specified a priori). Consistent with the previous LONDI evaluations [23,38], the study was grounded on 4 of the 5 RE-AIM dimensions: reach, adoption, implementation, and maintenance. The effectiveness dimension was not included, as this study is based on real-life use in nonexperimental settings. Therefore, measuring effectiveness (eg, the platform’s effect on children’s academic outcomes) was not feasible. This aligns with the study by Glasgow et al [20], whose recommendation is to only evaluate RE-AIM dimensions within a study’s scope. For the evaluated dimensions, four research questions were defined, with a particular focus on user groups of mental health professionals (ie, learning therapists and school psychologists). Specifically, the following research questions were formulated: (1) How many mental health professionals does LONDI reach (reach)? (2) Do mental health professionals intend to keep using the help system and why (adoption)? (3) In what manner do users implement pages intended for mental health professionals (implementation)? and (4) Does platform use change over time (maintenance)?

## Methods

### Overview

To answer the research questions, the study used a multimodal data collection process combining web analytics as well as self-reported demographic and UX measures.

### Data Collection

Data collection took place over the 10-month period between May 1, 2024, and March 1, 2025. During this time period, the LONDI platform was advertised on social media platforms targeting users in regions in which the majority of the population are native German speakers (ie, Germany, Switzerland, and Austria). Visits to the LONDI platform were tracked, and data were also collected from voluntarily completed pop-up questionnaires. The data collected for this study enabled an extension of the previous evaluations in 2 ways. First, by assessing the newest platform version (ie, launched in 2024), and second, by incorporating additional state-of-the-art research methods, namely, reading time and chatbot engagement.

### Measures

#### Overview

The specific measures used to evaluate each research question are detailed below. These measures were either self-reported or implemented using the web analytics (ie, data tracking) software Matomo (Figure S1 in Multimedia Appendix 1) [22]. Table 1 provides an overview of research questions, measures, and analyses.

**Table 1:** Summarized overview of research questions, measures, and analyses.

Research question	Measures	Analyses
Reach: how many mental health professionals (ie, learning therapists and school psychologists) does LONDI^a^ reach?	Demographics and professional experience	The relative percentage of mental health professionals using LONDI vs their number in the general population
Adoption: do mental health professionals intend to keep using the help system and why?	UX^b^ and reuse intention	Mean reuse intention ratings vs scale mean and vs mean score obtained in a previous evaluation [23] Mean UX ratings vs benchmarks Multiple linear regressions to assess if UX ratings predict reuse intention
Implementation: in what manner do users implement pages intended for mental health professionals?	Reading time and chatbot engagement	Mean time on pages vs minimum time needed to read them, calculated according to words per minute estimates Chatbot engagement rate vs benchmark
Maintenance: does platform use change over time?	Use comparisons	Number and location of users Time spent on the platform Number of actions per visit Devices and software

^a^Lernstörungen Online-Diagnostik und Intervention.

^b^UX: user experience.

#### Demographics and Professional Experience (Self-Reported)

To evaluate research question 1 (reach: How many mental health professionals does LONDI reach?), a pop-up questionnaire was used to collect demographic and professional data (Figure S2 in Multimedia Appendix 1). The questionnaire had 6 items and was programmed to appear on the front page of the platform. Participation was not mandatory, and users could opt out by closing the window in which the questionnaire appeared. The 6 items concerned users’ profession (ie, teacher, school psychologist, learning therapist, unemployed, or other), years of professional experience (ie, ranging from novice to more than 20 years), age, gender, professional qualifications, and whether they think that more knowledge in the field of LDs would be helpful in their day-to-day tasks. The percentage of learning therapists and school psychologists among the other professions was calculated and compared with their percentage in Germany.

#### UX and Reuse Intention (Self-Reported)

To evaluate research question 2 (adoption: Do mental health professionals intend to keep using the help system and why?), a pop-up questionnaire was used to collect UX and reuse intention data (Table S2 in Multimedia Appendix 1). The questionnaire had 10 items and was programmed to appear after using the help system. Participation was not mandatory, and users could opt out by closing the window in which the questionnaire appeared. Eight of the 10 items concerned UX, derived from the short version of the User Experience Questionnaire [40]. The items were presented using a 7-point semantic differential scale (eg, boring vs exciting). The first 4 adjective pairs measured the help system’s pragmatic qualities (ie, practical aspects), and the last 4 measured its hedonic qualities (ie, pleasurable aspects). Answers were coded in a range between –3 and +3 for the most negative and positive options, respectively. The comparison with the scale benchmarks was used to evaluate the ratings. One additional item concerned users’ profession, and the last item concerned intention to reuse (ie, “I plan to continue using LONDI”), derived from a validated questionnaire [41]. Possible answers appeared on a 7-point scale, with answers ranging from “do not agree at all” to “fully agree.” Answers to this item were also coded in a range between –3 and +3 for the most negative and positive options, respectively. As there are no benchmarks for this item, the scale mean was used to evaluate the ratings. Thus, ratings above or below the mean served as indicators of the intention to reuse the help system. Moreover, as these items were also used in the evaluation of the previous help system version, this study’s ratings were also compared with those obtained in the previous evaluation [23].

#### Reading Time (Tracked Data)

To evaluate research question 3 (implementation: In what manner do users implement pages intended for mental health professionals?), data pertaining to the average amount of time users spent on different LONDI pages were tracked. The reading time metric was chosen as a way to measure whether users spend enough time on the platform to read its content. As this study focused on mental health professionals, the time spent on the 5 pages intended for them was tracked. Of these 5 pages, 2 were index pages, and 3 were informational pages. Whereas the index pages did not contain a lot of words and their main purpose was to provide links directing users to other relevant pages, the informational pages contained detailed information divided into sections. For the index pages, the total number of words was counted. For the informational pages, the number of words per section was counted. To calculate expected reading time thresholds, the average German WPM rate for silent reading, which is 260 words per minute, was used [26]. Based on this, the minimum time needed to read each of the 5 pages was calculated, taking into account the number of words in the shortest section per informational page, and the total number of words per index page (Table S3 in Multimedia Appendix 1). The average time spent on the pages was compared with the minimum time needed to read them.

#### Chatbot Engagement (Tracked Data)

To evaluate research question 3 (implementation: In what manner do users implement pages intended for mental health professionals?), in addition to reading time, the ArtiBot.ai software [42] was used to track users’ engagement with a simple chatbot. The chatbot engagement metric was chosen as a way to measure whether users chose to interact with a virtual agent, and if this interaction exceeded 1 interaction. The chatbot was programmed as a frequently asked questions (FAQ) chatbot (Figure S3 in Multimedia Appendix 1). These types of chatbots operate in a question-answer manner, wherein users cannot type questions but are presented with options they can tap or click on [7]. Notably, this type of chatbot is only capable of answering a predefined set of questions with predefined answers (eg, “Would you like more information about…?” Then users can click “Yes” to get suggested options or “No” to end the chat). The chatbot appeared as a minimized chat bubble, and users could choose to open it by clicking or tapping it. The conversation turns per session (CPS) metric was used to assess user engagement [43]. CPS is the average number of conversation turns between a user and a chatbot. For this study, CPS was simplified due to the restricted nature of the FAQ chatbot. Engagement was counted when a visit contained the actions of opening the chat bubble and clicking or tapping on at least 1 offered option. As there are no benchmarks in the context of digital health, the comparison with industry benchmarks was used to evaluate the chatbot engagement rate (ie, successful engagement rates range around 35%-40% [44]).

#### Use Comparisons (Tracked Data)

To evaluate research question 4 (maintenance: Does platform use change over time?), the Matomo analytics software [22] (Figure S1 in Multimedia Appendix 1) was used. For this purpose, the Matomo date comparison feature was activated. This feature enables the comparison of date ranges for any Matomo report containing dates as a dimension. The compared time periods were the 10-month period of this study and the 10-month period of the prior year (ie, before the latest platform revisions were made). Specifically, the following parameters were compared: number of visits, user locations, average time spent on the platform, times of the day in local time with most visits, average number of actions per visit (ie, average number of interactions, including page views, downloads, and links clicked per single visit), and visits per device and software type. As there are no benchmarks for user growth rate in the context of digital health, the comparison with industry benchmarks was used (ie, it is typically viewed positively when the monthly growth in the number of website visits is 10%-20% for a new website, and 5%-10% for established websites [45]).

### Ethical Considerations

This study was part of a larger project (ie, LONDI), approved by the ethics committee at the university hospital of the Ludwig Maximilian University of Munich (approval number 22-0300 1 V). Participants who took part in the project’s controlled experimental studies provided written informed consent. This was not done for this study, due to its real-world setting. Namely, its participants were those who used the online platform of their own volition, rather than participating in a study done in an experimental setting. Given these circumstances, the ethics committee approved waiving informed consent for this study, provided that strict measures to ensure anonymity were adhered to. Specifically, users’ IP addresses were anonymized via masking by Matomo, in accordance with strict data protection laws, and the experimenters had no access to participants’ unmasked IP addresses or any other identifying data. Furthermore, when accessing the platform, all users were informed via a pop-up notice that the website uses cookies and similar technologies to track data and were given the option to accept or opt out. Compensation was not offered.

## Results

The overall sample comprised 37,133 tracked platform visits. The visit bounce rate (ie, proportion of visitors that left the website after only viewing 1 page) was 18.99% (7055/37,133). The tracked data from the overall sample revealed that the majority of visits were from Germany. More specifically, although visits originated from 113 distinct countries, 77.32% (28,712/37,133) were from Germany. This is in line with the fact that LONDI is currently only available in German. However, the second, third, and fourth locations with the most visits were the United States, Canada, and Ireland (1122/37,133, 3.02%; 798/37,133, 2.15%; 771/37,133, 2.08%, respectively). Among the 16 German states, the majority of visits (16,436/37,133, 44.26%) were from the state of Hesse, followed by 10.47% (3891/37,133) from North Rhine-Westphalia, 5.63% (2094/37,133) from Bavaria, 5% (1854/37,133) from Berlin, 3.1% (1164/37,133) from Baden-Württemberg, and 2.8% from Lower Saxony (1032/37,133).

To answer research question 1 (reach: How many mental health professionals does LONDI reach?), users’ answers to the first pop-up questionnaire (n=1324), collecting demographic and professional experience, were analyzed. Among the users who filled out the questionnaire, 21.90% (291/1324) stated that they were learning therapists, and 10.64% (141/1324) stated that they were school psychologists, with various years of experience (Table S4 in Multimedia Appendix 1). Although the exact number of learning therapists is not publicly known, according to a representative from the German Association for Dyslexia and Dyscalculia, the combined number for Germany and Austria is roughly 2500 (L Weinreich, personal correspondence, February 23, 2023). The number of school psychologists in Germany is publicly known and is roughly 2,500 as well [46]. Based on these estimates, with an adult population of more than 70 million [47], their percentage in the German population is lower than 0.01%. As the relative percentage of mental health professionals using the platform was higher than that in the general population, it can be deduced that the platform reached this user group.

**Figure 1:**
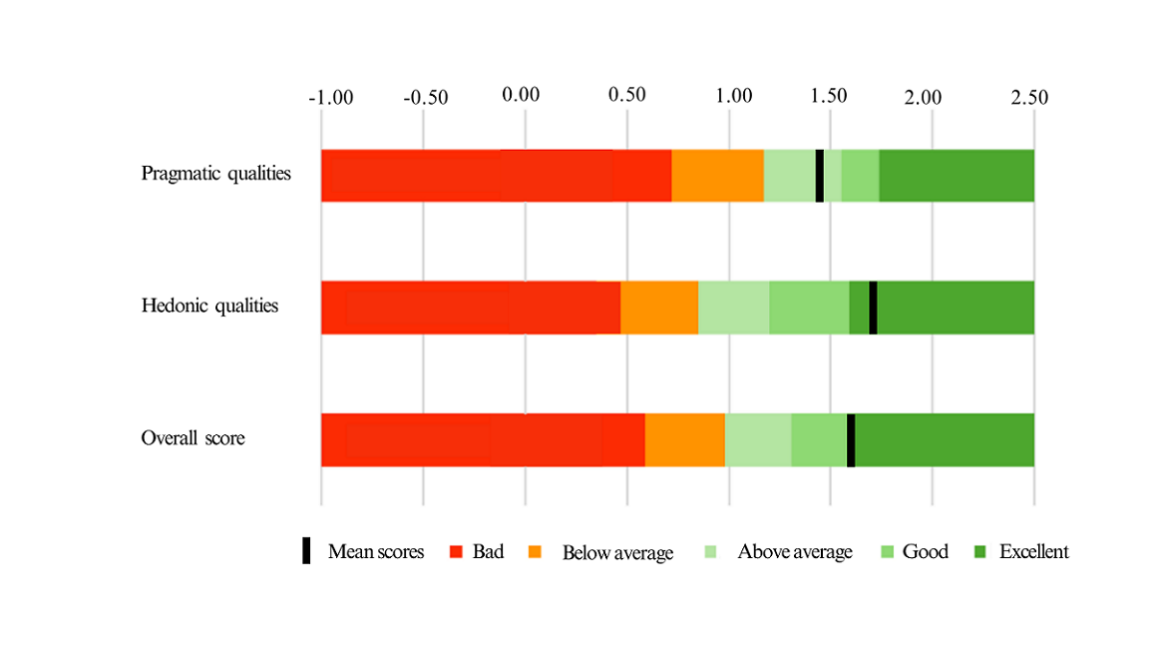
Mental health professionals’ mean user experience (UX) scores for the help system. The scores are depicted within the short version of the User Experience Questionnaire benchmark range.

To answer research question 2 (adoption: Do mental health professionals intend to keep using the help system and why?), mental health professionals’ answers to the second pop-up questionnaire (n=160), measuring UX and reuse intention for the platform’s help system, were analyzed. This sample size was smaller than that of the first pop-up questionnaire, likely since the second pop-up questionnaire only appeared to users who completed all the steps required to use the help system (ie, answering all the questions regarding a child’s learning profile). The items’ mean scores were 1.54 (SD 1.14) for all items combined, 1.45 (SD 1.28) for the pragmatic qualities, and 1.64 (SD 1.14) for the hedonic qualities. According to the short version of the User Experience Questionnaire benchmarks [48], these scores can be interpreted as above average for the pragmatic qualities, good for all items combined, and excellent for the hedonic qualities (Figure 1; Hinderks et al [48]). For the item assessing intention to reuse, the mean score was 2.02 (SD 1.21), which is higher than the scale mean (ie, 0) and higher than the ratings obtained in the evaluation of the previous help system version (ie, 1.90). Additionally, 2 linear regressions were performed to assess whether the different UX qualities predict intention to reuse. Models for items assessing both qualities were significant for pragmatic qualities (*F*_1,158_=26.47; *P*<.001), accounting for 14% of the variance, and for hedonic qualities (*F*_1,158_=32.57; *P*<.001), accounting for 17% of the variance. Thus, it can be deduced that mental health professionals planned to adopt the help system.

To answer research question 3 (implementation: In what manner do users implement pages intended for mental health professionals?), tracked data measuring both reading time and chatbot engagement were analyzed. Reading time data revealed that the average time users spent on the tracked pages was lower than the minimum time required to read at least 1 complete page section (Table 2). This was the case for both the index (ie, pages designed to redirect users) and the information pages (ie, pages containing detailed information). Furthermore, chatbot engagement data revealed that users rarely interacted with the chatbot. In fact, only 0.18% (67/37,133) of the total number of platform visits included chatbot engagement. This rate is far below the desired engagement level of at least 35% [44]. Thus, it can be deduced that the platform’s implementation was suboptimal.

**Table 2:** Time spent on pages for mental health professionals vs the minimum reading time.

Page	Mean time on page (seconds)	Minimum time to read^a^ (seconds)
Index for learning therapists	16	18.69
Index for school psychologists	12	26.77
Inf. for learning therapists	17	60.23
Inf. for school psychologists 1	12	72.00
Inf. for school psychologists 2	18	60.91

^a^Minimum reading time was based on the average German word-per-minute rate for silent reading [26]. As the information pages contained multiple sections, the shortest section for each page was used.

To answer research question 4 (maintenance: Does platform use change over time?), tracked data assessing maintenance over 2 time periods were analyzed (Table 3). Notably, the number of visits increased by more than 80% (a visualization of visits over time and a comparison of platform traffic sources in 2 time periods is available in Figures S4 and S5 in Multimedia Appendix 1, respectively). This growth is largely related to social media campaigns, accounting for 53% (19,741/37,133) of the user traffic in the second time period, vs less than 1% in the first time period (n=2). Interestingly, in both time periods, none of the user traffic was accounted for by AI assistants (visits from tools such as ChatGPT, Microsoft Copilot, Claude). Since an 80% increase over 1 year is the equivalent of a 5.02% monthly increase, this is a good rate, as desired monthly growth rates should exceed 5% [45]. Thus, it can be deduced that platform use changes over time, in a manner that can be perceived as favorable (ie, increased web traffic). Nevertheless, it is not possible to deduce how many of these visits were by new vs returning users, as users’ IP addresses were masked.

**Table 3:** Comparison of platform use in the 2 time periods.

Category and subcategory		T1^a^	T2^b^	Difference, %
Total visits, n		20,496	37,133	+81.2
**Visits at frequent locations, n**				
	Germany	16,119	28,712	+78.1
	United States	395	1,122	+184.1
	Canada	279	798	+186
	Ireland	411	771	+87.6
	Austria	415	608	+46.5
	United Kingdom	216	600	+177.8
	Netherlands	243	513	+111.1
	Switzerland	288	449	+55.9
Mean visit duration		1 minute 46 seconds	1 minute 44 seconds	–1.9 seconds
Mean actions per visit, n		3.2	10.1	+215.6
**Frequently used devices, n**				
	Smartphone	14,490	26,298	+81.5
	Desktop	5,508	10,200	+85.2
	Tablet	351	433	+23.4
**Visits by operating system family, n**				
	Android	8,344	14,229	+70.5
	iOS	6,508	12,622	+93.9
	Windows	3,561	6,101	+71.3
	Mac	1,723	3,753	+117.8

^a^The time period between May 1, 2023, and March 1, 2024.

^b^The time period between May 1, 2024, and March 1, 2025.

## Discussion

### Principal Results

This study’s goal was to perform an assessment of a digital health platform, using both web analytics and UX measurements. Specifically, the following 4 RE-AIM framework dimensions were assessed: reach, adoption, implementation, and maintenance. In terms of reach and adoption, the study results revealed that the platform reached its target population of mental health professionals and that they plan to adopt it in their everyday practice. Moreover, users rated the platform’s help system above the benchmark average both in terms of its pragmatic and its hedonic qualities. In terms of implementation, the results revealed that users did not implement the platform as expected, as indicated by the average time they spent on it and by the low chatbot engagement rate. In terms of maintenance, the results revealed that platform use changed over time, predominantly indicated by the sharp increase in the number of visits. All in all, the results from all but one of the assessed RE-AIM dimensions, namely, implementation, reflected a positive public health contribution.

The assessed metrics for reach and adoption were, respectively, the number of mental health professionals among the LONDI users, and their intention to keep using its help system. Results for the reach dimension revealed that the proportion of both user groups of mental health professionals (ie, learning therapists and school psychologists) exceeded their proportion in the German population. In the evaluation study of the initial version of LONDI [23], it was also found that the platform reached targeted users. This study’s results demonstrate that the retention was sustained. This is promising, as there are other valuable sources in the context of LDs for German speakers that users could have reverted to [49]. Nevertheless, the number of learning therapists was more than double that of the school psychologists, even though their estimated number in the German population is comparable. Further marketing endeavors are required to ensure that the platform is advertised equally to all its user groups. The adoption results revealed that users intend to keep using the help system, and this was predicted by UX qualities (eg, ease of use). Users’ intention to reuse the help system increased compared to the first phase evaluation [23]. This points toward the potential positive contribution caused by revising the help system (ie, the design was simplified and instructional tutorials were created). Continued endeavors are required for technical maintenance of the help system, as well as regular updates of the featured diagnostic and intervention recommendations. Thus, results from both the reach and adoption metrics were positive, and further endeavors are needed to optimize and retain this.

The assessed metrics for implementation were the reading time of the pages intended for mental health professionals, as well as chatbot engagement. These metrics were not used in the first LONDI evaluation phase. Rather, they were chosen based on insights from a first phase study, which used popular metrics used in commercial websites [23]. For example, the first phase study used the conversion rate metric (ie, the percentage of website visitors who completed a desired goal) to assess the platform’s implementation. However, such metrics were found to be suboptimal in the context of digital health, as their interpretation is based on online marketing benchmarks for profit-driven websites [50]. In this study, the chosen implementation metrics assessed whether users spend enough time on the platform to read its content and whether they interact with its chatbot.

The reading time results revealed that the average time users spent on the pages intended for mental health professionals was lower than the minimum time required to read them. The time users spend on pages is an important indicator of users’ engagement with platform content, as longer sessions enable more in-depth reading [27]. Notably, in the first LONDI evaluation phase, a notable shift occurred over time, with more users accessing the platform via smartphones rather than desktop devices [23]. This was also the case for the revised LONDI version, with this study revealing that most users accessed the platform via smartphones. To optimize users’ smartphone experience, the design of the revised LONDI version was optimized for smartphone use. Additionally, in line with the recommendation to improve online readability by designing pages with low visual complexity [51], infographics were added to break up long text blocks. Nevertheless, on average, users did not spend enough time on the tracked pages to read their content. Paradoxically, despite the rise in average screen times in recent years, which would, in theory, allow users to have longer reading sessions, smartphones are associated with reduced reading durations [52]. It is possible that LONDI visitors used smartphones for superficial reading of the platform’s content (eg, only reading the headings). This is in line with other studies demonstrating that compared to printed texts, online texts are read in a quicker, shallower manner, leading to reduced reading comprehension [53-55]. Thus, the findings from this study could be taken as an indication of low user engagement. Alternatively, it is possible that users wanted to get a quick overview of the platform, with the intention of reading certain content in more detail at a later point.

Notably, in compliance with GDPR, users’ data were anonymous (users’ IPs were masked), making it impossible to distinguish new users from returning users. Previous studies have shown that returning users need less time to read digital content [56]. A future assessment could ask users to use individual trackable links, enabling a distinction between the use data of new vs returning users. Interestingly, the reading times of all the tracked pages, regardless of their word count, ranged between 12 and 18 seconds. A future assessment could also assess whether there is a ceiling effect (ie, the maximum amount of time users spent on pages) and whether this differs between new and returning users.

In addition to reading time, the other assessed implementation metric was chatbot engagement, revealing an engagement rate far below the range suggested by industry benchmarks [44]. However, this finding needs to be interpreted cautiously, as the comparison relied on industry benchmarks due to the lack of established standards for digital health chatbots. Moreover, the LONDI platform was not designed for profit, unlike many industry-based platforms. Thus, this study’s comparison with industry benchmarks should be viewed as exploratory. In line with the findings of Abd-Alrazaq et al [39], further research is required to establish standardized measures for chatbot engagement in the digital health context. Furthermore, in this study, a simple FAQ chatbot was chosen over a more complex chatbot, as its implementation was feasible within the study’s scope. The results revealed that chatbot engagement rates were far below the industry benchmark [44]. One possible reason for the low engagement could be that the users did not think the chatbot was useful, as it only offered predefined questions and answers, not allowing users to type their own questions. This is in line with the notion that, despite the spike in technological advancements, many online interactions are still clumsy and do not fulfill their intended purpose [1]. Further endeavors are required to provide a more complex chatbot, better simulating a conversation with a human agent. Another reason for the low engagement could be related to users’ trust in AI and their willingness to engage with chatbots. Most of the visits to LONDI occurred from Germany. Interestingly, a study by Chang et al [57] showed that compared with other nationalities (eg, Brazilians), Germans are less open to engaging with chatbots. Another reason for the low engagement could be that the chatbot was too hidden, namely, users did not notice the chat bubble icon they had to press for the chatbot to open. This was done in order not to aggravate the users by having the chatbot pop up whenever they use LONDI. This is in line with the literature showing that users prefer a “polite pop-up” that only opens when clicked on [58]. Thus, future research should qualitatively examine user reactions to a more complex, attention-grabbing chatbot.

The assessed metric for maintenance was the use comparison between the previous and the revised platform versions. One notable result was that the number of visits increased by roughly 81%. This is encouraging, as increasing user traffic increases a website’s impact [59]. Nevertheless, this finding should be interpreted cautiously, as it stands in contrast with the finding that, on average, users do not spend enough time on the platform to read its content. Taken together, these results suggest that while visits to the platform increased, many visits were brief and allowed only limited, surface-level engagement. Importantly, the growth in the number of visits was largely accounted for by social media campaigns (ie, the LONDI Instagram and Facebook accounts). This stresses the importance of instrumentalizing social media to attract visitors. Notably, this growth was entirely not accounted for by referrals from AI assistants. This could be the result of the period of data collection ending in early 2025. European-wide data show that adults used AI less frequently than adolescents and young adults in 2025 [60]. To examine if these changes occur in the future, further endeavors should be made to examine changes in AI assistant referrals over time. Moreover, the written content of the revised platform version does not differ from the content of the previous version. Rather, the versions differ in their design (ie, the new version is more suitable for smartphone use, features more infographics, and the help system’s design was simplified, and instructional tutorials were added). This stresses the importance of creating a user-friendly design to attract visitors.

Another notable result was the increase in the number of visits from the United States, Canada, and Ireland, even though the platform’s content is entirely in German. This is surprising, as the number of users from these countries exceeded the number of users from Austria and Switzerland, where German is an official language [61]. One explanation for this could be that users were using a virtual private network, which can make it seem as if users are using the platform from a different location [62]. Another explanation could be that the platform was read in different languages using machine translation (eg, the Google Translate browser extension). Machine translation is often favored over professional translation, the latter being much more resource-intensive and time-consuming [63]. However, machine translation is not suitable for the LONDI platform, as a lot of its content is tailored to the needs of German residents (eg, specific information on the German school system). Thus, further endeavors should be made to maintain the growth in user traffic and to obtain the resources to professionally translate and adapt its content to other languages.

### Limitations

This study is not without its limitations. One limitation is inherent in the use of web analytics. While web analytics offer valuable insights, it is not possible to use them to infer actual engagement with platform content or users’ reading comprehension. In particular, the results concerning reading times and chatbot engagement need to be interpreted with caution, especially since there are no standard benchmarks for chatbot engagement in the context of digital health. As the purpose of the assessment was to evaluate how users use the platform in a natural setting, the sample consisted of anonymous users using the platform of their own accord. Therefore, it was not possible to use this sample to gather qualitative data (eg, via interviews) or to follow up with individual users longitudinally. Nevertheless, the study did use other quantitative measures (ie, pop-up questionnaires) to collect user input. Moreover, as the study was part of a larger project, it is complemented by another preregistered study that uses qualitative measures [38]. A second limitation is the possibility of a self-selection bias. When participants voluntarily participate in a study, they are likely to have similar attitudes and traits, compromising sample diversity [64]. On the one hand, this was not a crucial point for this study, as its goal was to target a very specific population, namely, German speakers who have a special interest in supporting children with LDs. On the other hand, this limits the findings’ external validity. Therefore, further similar evaluations targeting different populations are needed, particularly for the interpretation of the RE-AIM adoption and reach dimensions. A third limitation is the smaller number of participants (n=160) used to evaluate the adoption dimension, likely due to the smaller number of participants completing the help system process and answering the respective questionnaire. While this limits this dimension’s generalizability, the other dimensions were measured with larger sample sizes (eg, N=37,133 for implementation), contributing to the current study’s strength. A fourth limitation is that the platform’s impact on children’s academic outcomes was not measured (ie, the RE-AIM effectiveness dimension). A recent systematic review has shown that psychoeducational LD interventions targeting adults, namely parents, are associated with improvements in children’s academic, behavioral, and social outcomes [65]. Thus, a future study, with a different methodological and analytical focus, could assess effectiveness by recruiting families and conducting premeasurements and postmeasurements.

### Conclusions

While the influx of digital health brings many benefits, it also increases the risk of misinformation and misconceptions. Digital health tools developed by professionals and academic institutions can potentially combat these risks, but they must be rigorously assessed. This study assessed the LONDI platform, based on the following 4 dimensions of the RE-AIM framework: reach, adoption, implementation, and maintenance. Results from both web analytics and UX measurements indicated a positive public health contribution in all but the implementation dimension. Specifically, users did not spend enough time on pages to read their content and did not engage with the platform’s chatbot as expected. Standard benchmarks for engagement with digital health chatbots are needed. Further research is needed to verify if reading times depend on whether users are new or returning, if there is a ceiling effect for time spent on pages, and if a more complex chatbot will lead to more engagement. As digital health continues to expand, more assessment studies are essential to ensure these tools perform as intended.

## Data Availability

The datasets generated and analyzed during this study are available from the corresponding author on reasonable request.

## Appendix

Multimedia Appendix 1 Supplementary tables and figures detailing research design, user demographics, experience questionnaires, estimated reading times, and software screenshots.

## Abbreviations

AI: artificial intelligence
CPS: conversation turns per session
FAQ: frequently asked question
GDPR: General Data Protection Regulation
LD: learning disorder
LONDI: Lernstörungen Online-Diagnostik und Intervention
RE-AIM: reach, effectiveness, adoption, implementation, and maintenance
UX: user experience
WPM: words per minute
