# Supplementary material for: Assessment of a Digital Health Platform Using Web Analytics and User Experience Measurements: Quantitative Study Based on RE-AIM
Source: J Med Internet Res. 2026 Jul 2;28:e64903. doi: 10.2196/64903 (PMC13376844; doi:10.2196/64903)
Supplement: Multimedia Appendix 1 [file jmir_v28i1e64903_app1.docx]

# Multimedia Appendix 1

## Table S1

### A-priori specified elements

| Dimension | Research question | Methods | Analyses |
| --- | --- | --- | --- |
| Reach | How many mental health professionals (i.e., learning therapists and school psychologists) does LONDI reach? | Users’ profession, as reported in an optional demographic pop-up questionnaire that appears on the front page of londi.de. | The relative percentage of mental health professionals using LONDI will be compared with that of the general population. |
| Adoption | Do mental health professionals intend to keep using the help system and why? | Mental health professionals’ answer to the item: “I plan to continue using Londi” (1 = Do not agree at all to 7 = Fully agree) and to the short version of the User Experience Questionnaire (UEQ-S) (-3 = a negative term to 3 = the opposite positive term).  These are featured in an optional pop-up questionnaire that appears after using the LONDI help system. Answers are on a 7-point scale. | Mean answers for the item (1 to 7) and for the UEQ-S (-3 to 3).  The item will be assessed based on whether the score mean is higher or lower than the average, and the UEQ-S will be compared with existing benchmarks.  Multiple linear regressions will be performed to assess whether the help system’s hedonic and pragmatic qualities predict the answer to the item “I plan to continue using LONDI”. |
| Implementation | In what manner do users implement pages intended for mental health professionals? | Time goals predefined as the minimum time that needs to be spent on the pages intended for mental health professionals for them to be counted as read. Users’ average time on pages will be measured in Matomo, then compared with time goals, calculated according a German word per minute estimate (Brysbaert, 2019).  Chatbot engagement, as measured by the click rate on an optional “chat bubble” that users can open from any page of londi.de. High engagement will be indicated by a click rate > 40% (Behavioural Insights Team, 2023). | Data on the average time spent on the pages intended for mental health professionals will be collected, and compared with time goals.  The number of users that open the “chat bubble” will be analyzed, as well as the percentage of users continuing the engagement/closing the chatbot immediately afterwards. Engagement rates will be compared with rates from a large-scale study on chatbot engagement in public services (Behavioural Insights Team, 2023). |
| Maintenance | Does platform usage change over time? | Usage in 01.05.23 - 01.08.23 vs. 01.05.24 - 01.08.24 for all users. These time segments were chosen as the new optimized version of LONDI was launched in 2024, and the Matomo data collection for this version began on 15.04.24. Each time period represents the same three-month period in different years (i.e., 2023/2024), in either the previous or optimized londi.de version. | The following comparisons will be performed with Matomo:   - Number of users - Users’ locations - Time spent on the platform in general and on specific pages - Number of actions - Times of the day in which the platform was being used - Devices and software used |

*Note.* This table was originally published as part of a preregistration in the Open Science Framework (associated project link: https://osf.io/8fm2x).

## Figure S1

###
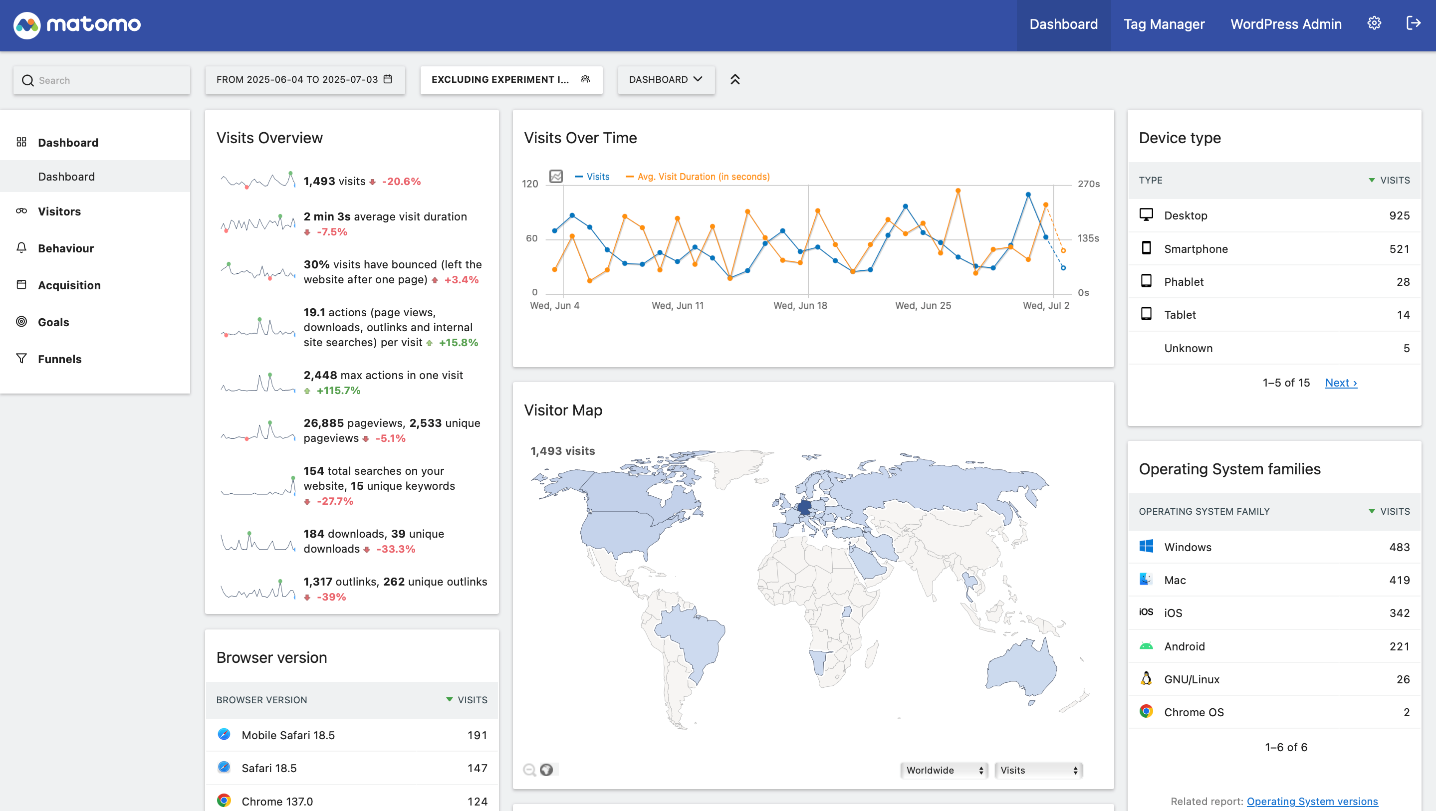
Screenshot of the Matomo Analytics Software

## Figure S2

### Screenshot of the demographic pop-up questioner

*
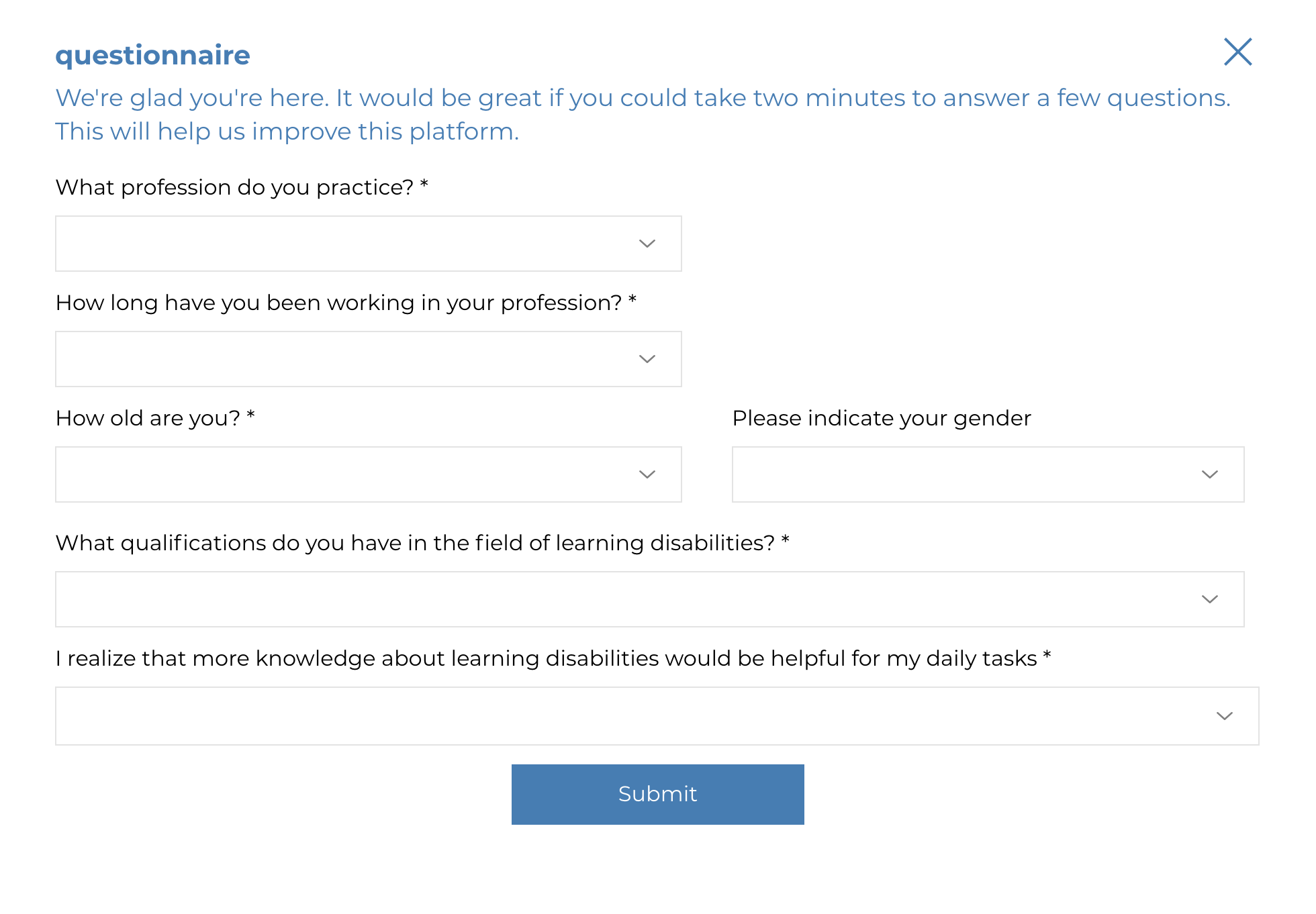
*

*Note.* All self-reported measures appearing in this appendix were originally in German, and have been translated into English to accommodate non-German speakers.

## Table S2

### Items included in the UX questionnaire

| Negative item | Rating | Positive item |
| --- | --- | --- |
| obstructive | o o o o o o o | supportive |
| complicated | o o o o o o o | easy |
| inefficient | o o o o o o o | efficient |
| confusing | o o o o o o o | clear |
| boring | o o o o o o o | exciting |
| not interesting | o o o o o o o | interesting |
| conventional | o o o o o o o | inventive |
| usual | o o o o o o o | leading edge |

*Note.* Items were taken from the short version of the User Experience Questionnaire (UEQ-S; Schrepp et al., 2017). Two additional items appeared in this questionnaire. One item asked users what their profession was. Possible answers were: teacher, school psychologist, learning therapists, no profession / unemployed, other. The other item asked users if they intend to continue using LONDI. Possible answers were: (1) yes, I fully agree, (2) yes, I agree, (3) I tend to agree, (4) neutral, (5) I tend to disagree, (6) no, I do not agree, (7) no, I do not agree at all.

## Table S3

### Estimated Reading Time for Different Informational Sections

| Page | Word Count | Min. Time to Read |
| --- | --- | --- |
| Index for learning therapists | 81 | 18.69 |
| Index for school psychologists | 116 | 26.77 |
| Inf. for learning therapists |  |  |
| section 1 | 1315 | 303.46 |
| section 2 | 278 | 64.15 |
| section 3 | 310 | 71.54 |
| section 4 | 510 | 117.69 |
| section 5 | 280 | 64.62 |
| section 6 | 261 | 60.23 |
| Inf. for school psychologists 1 |  |  |
| section 1 | 312 | 72.00 |
| section 2 | 1287 | 297.00 |
| section 3 | 702 | 162.00 |
| section 4 | 992 | 228.92 |
| Inf. for school psychologists 2 |  |  |
| section 1 | 264 | 60.91 |
| section 2 | 1267 | 292.38 |
| section 3 | 601 | 138.69 |
| section 4 | 930 | 214.62 |

*Note.* The minimum time needed to read each of the five pages was calculated based on the average German word per minute rate for silent reading, which is 260 words per minute (Brysbaert, 2019). For each of the informational pages, the minimum time was calculated separately for each section.

## Figure S3

### Screenshot of the LONDI homepage with an opened chatbot window

*
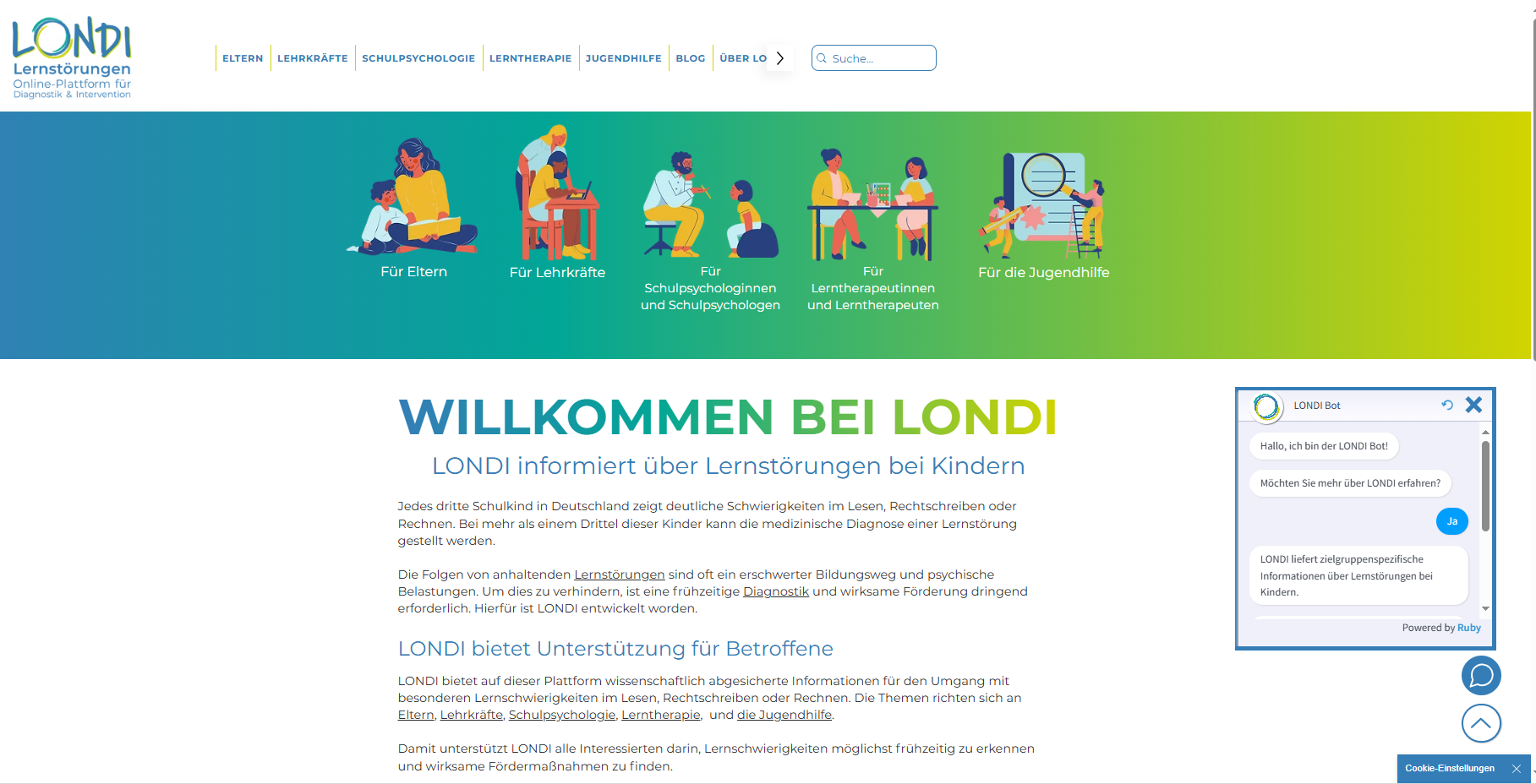
*

*Note.* The chatbot opens when users press the chat bubble icon, which appears on the bottom right corner.

## Table S4

### Years of Experience Among Learning Therapists and School Psychologists

| Years of Experience | Learning Therapists (n) | Learning Therapists (%) | School Psychologists (n) | School Psychologists (%) |
| --- | --- | --- | --- | --- |
| None | 21 | 7% | 12 | 9% |
| 1–5 | 96 | 33% | 25 | 18% |
| 6–10 | 54 | 19% | 33 | 23% |
| 10–20 | 68 | 23% | 48 | 34% |
| >20 | 52 | 18% | 23 | 16% |

## Figure S4

### Screenshot of the Matomo comparison of platform visits in two time periods


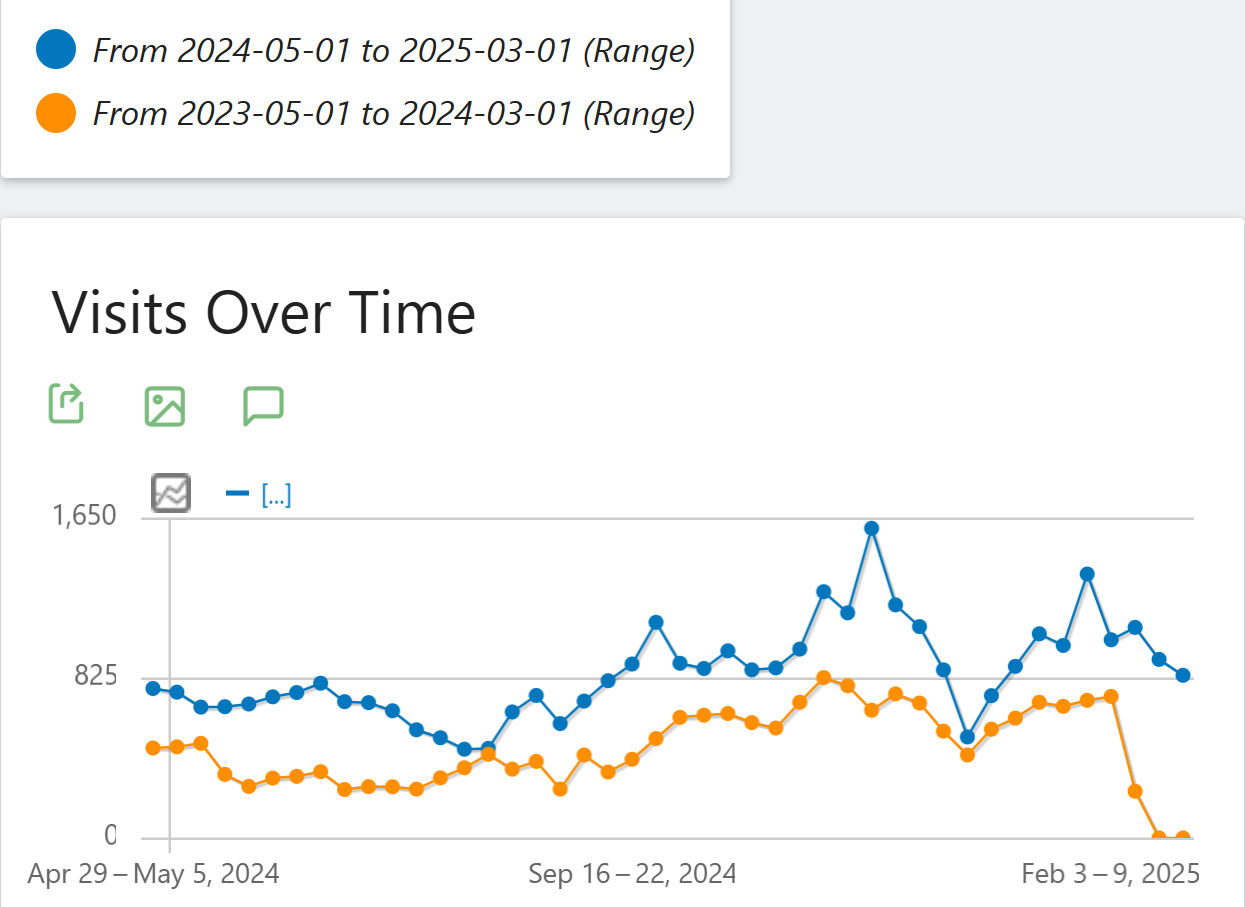


## Figure S5

### Screenshot of the Matomo comparison of platform traffic sources in two time periods


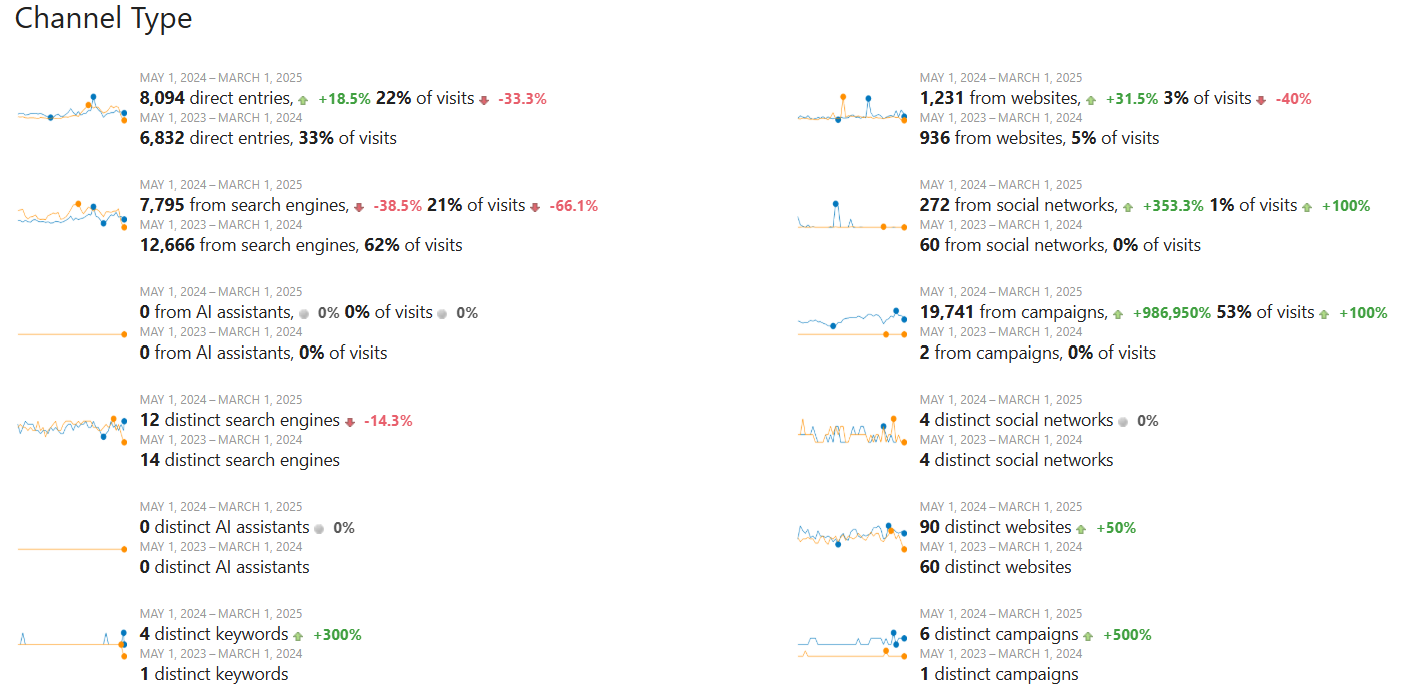


*Note.* Due to IP masking and the anonymization of users’ data, the traffic sources for some users are unavailable.
